# Supplementary material for: Laser-mediated rupture of chlamydial inclusions triggers pathogen egress and host cell necrosis
Source: Nat Commun. 2017 Mar 10;8:14729. doi: 10.1038/ncomms14729 (PMC5353685; doi:10.1038/ncomms14729)
Supplement: Supplementary Information — Supplementary Figures [file ncomms14729-s1.pdf]

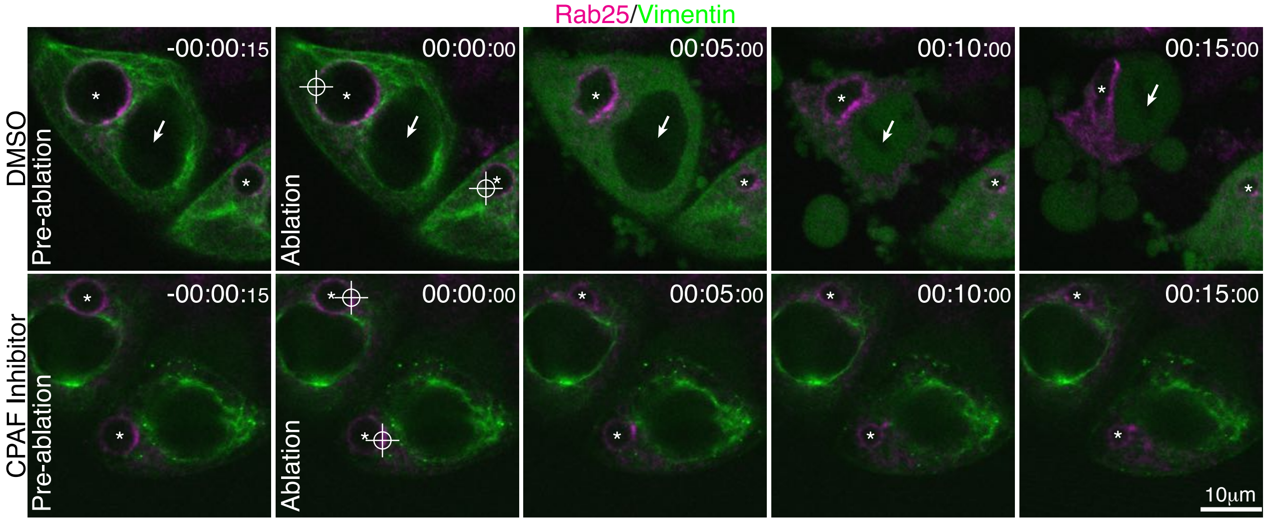

Supplementary Figure 1. Laser-mediated inclusion rupture triggers CPAF-dependent disruption of intermediate filaments. Time-lapse videomicroscopy of 2xGFP-vimentin-expressing mCherry-Rab25 stable HeLa cells infected for 24 h p.i. with CTL2 (MOI~0.5) and ablated in the presence and absence of a CPAF-inhibitory peptide. Arrows highlight the nuclei and asterisks the inclusion.

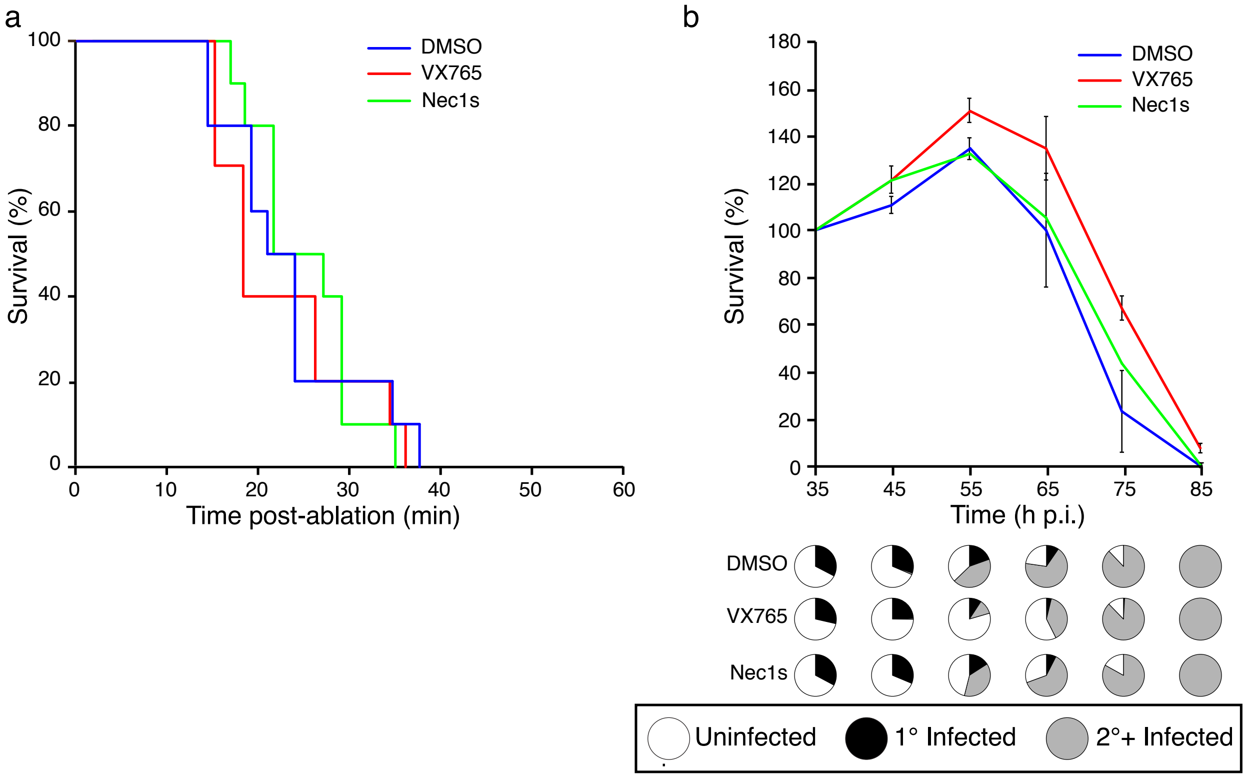

Supplementary Figure 2. Chlamydia-induced cell death is not disrupted by inhibition of RIP1 or caspase 1. a) Quantification of inclusion-rupture induced cell death under the indicated conditions. N=10 biological replicates for each condition. **b)** Quantification of native egress-induced cell death under the indicated conditions. Presented in pie-charts are the proportions of primary infected, ≥secondary infected and uninfected cells surviving cells at the indicated time-point as monitored by GFP-CTL2 fluorescence. N=3 biological replicates with >200 cells counted per replicate. Error bars present the Standard Deviation from the Mean. For clarity, only error bars for DMSO and VX765 are presented. *, $P \leq 0.05$; **, $P \leq 0.01$; ***, $P \leq 0.001$ (Unpaired Student's t-test).

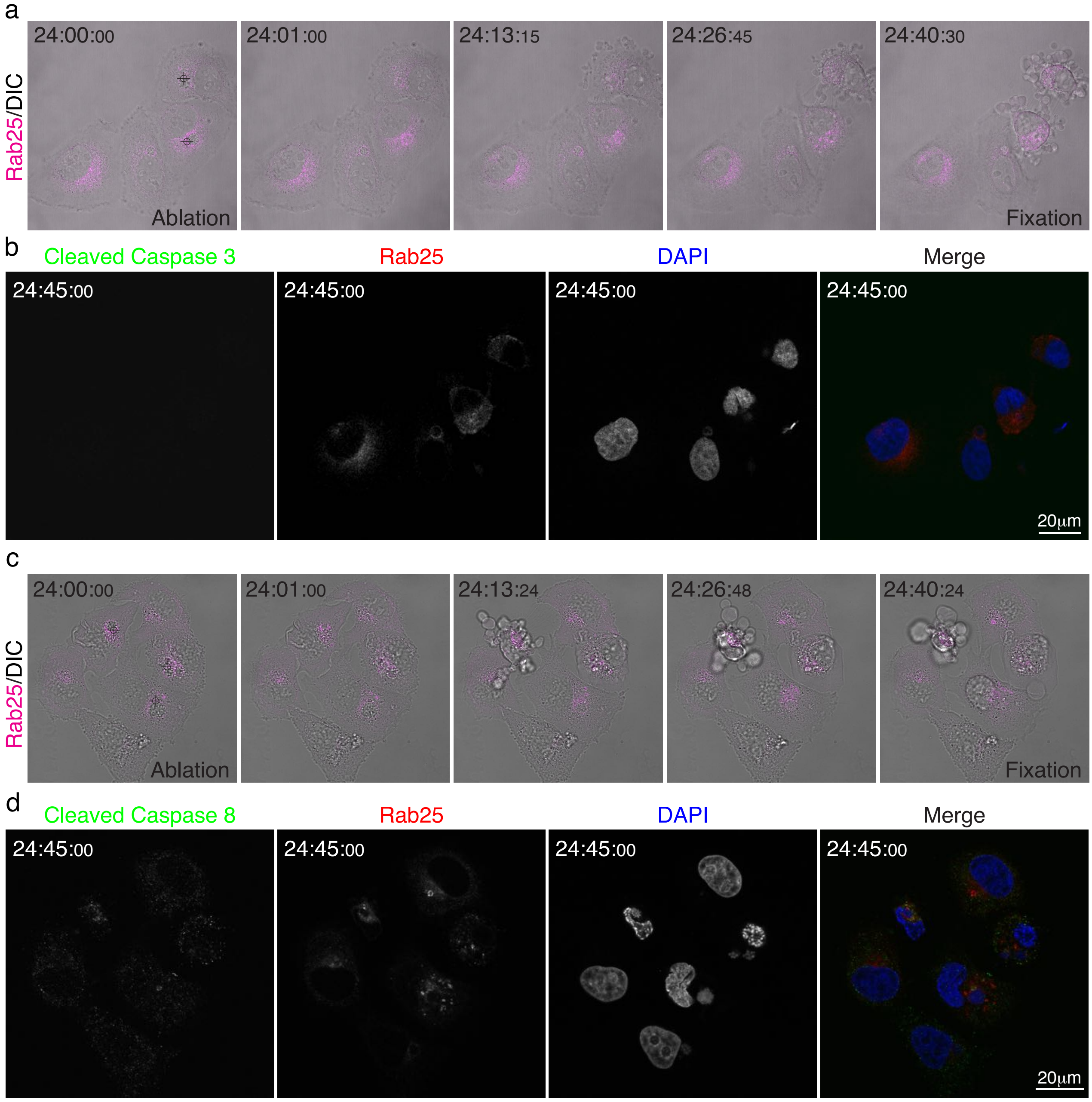

Supplementary Figure 3. Laser-mediated inclusion rupture does not activate caspase 3 or caspase 8. a) Time-lapse videomicroscopy of mCherry-Rab25 stable HeLa cells ablated 24h p.i. with CTL2 (MOI~0.5). **b)** Immunofluorescence of ablated cells with a cleaved caspase 3 antibody and DAPI. **c)** Time-lapse videomicroscopy of mCherry-Rab25 stable HeLa cells ablated 24h p.i. with CTL2 (MOI~0.5). **d)** Immunofluorescence of ablated cells with a cleaved caspase 8 antibody and DAPI.

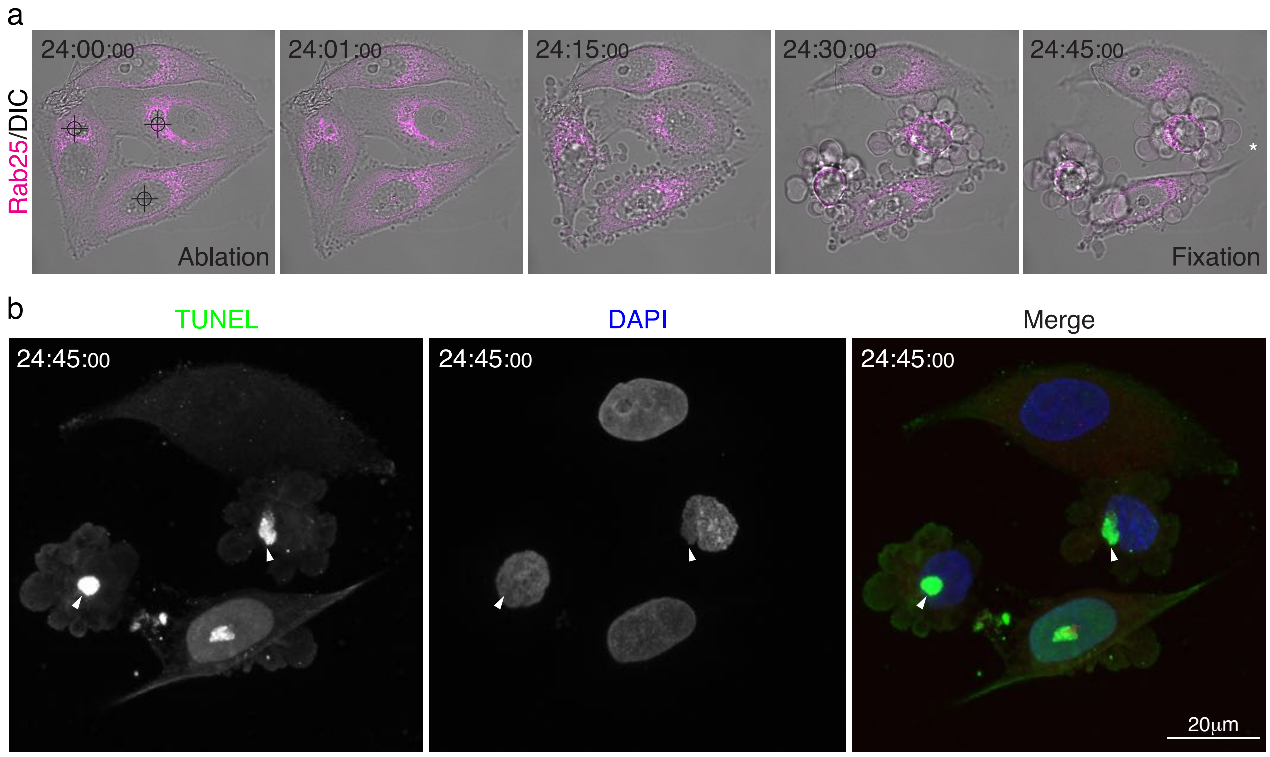

Supplementary Figure 4. Laser-mediated inclusion rupture does not initiate DNA fragmentation. a) Time-lapse videomicroscopy of mCherry-Rab25 stable HeLa cells ablated 24h p.i. with CTL2 (MOI~0.5). **b)** Maximum intensity projection of TUNEL-labelling of ablated cells counterstained with DAPI. Arrow heads highlight TUNEL-positive *C. trachomatis*.

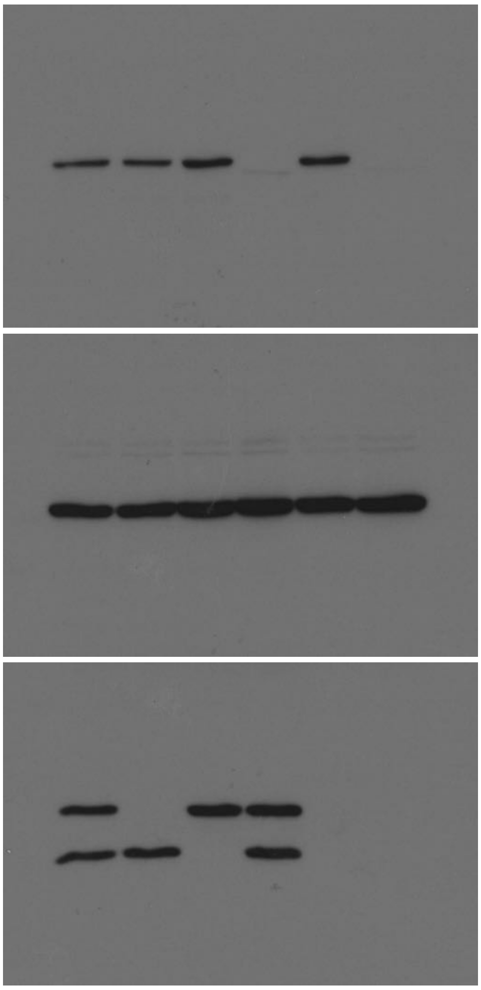

RIP1 (D94C12)
CST#3493
1:1000

β-tubulin
LI-COR Bioscience #926-42211
1:2000

BAK (D4E4)
CST#12105
1:1000

BAX (D2E11)
CST#5023
1:100

Supplementary Figure 5. Full scans of all blots presented in cropped form in figure 3d.

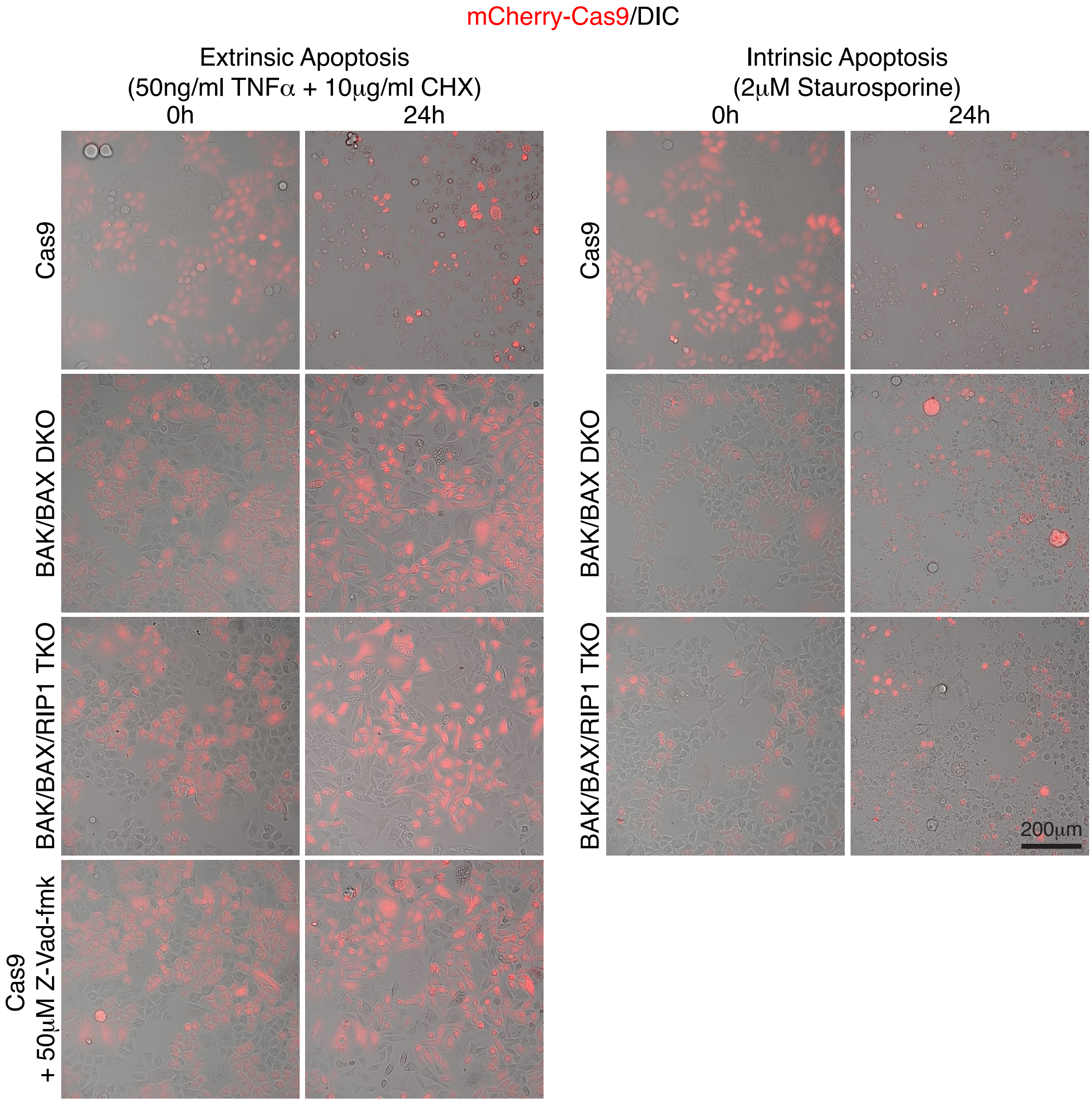

Supplementary Figure 6. BAK, BAX and RIP1 Double and Triple Knockouts are resistant to apoptosis. Live images of genome-edited HeLa cells following extrinsic and intrinsic apoptotic stimuli at the indicated time points.
